# Cases of Interstitial Tubo-Gestation

**Published:** 1883-05

**Authors:** 


CASE OF INTERSTITIAL TUBO-GESTATION.

Dr. Henry Habgood describes the case of a married woman, aged 35, who died with all the symptoms of internal hæmorrhage, in the eleventh week of pregnancy. "At the necropsy, there were about five pints of clotted blood in the pelvic and abdominal cavities. On turning this out, the source of the hæmorrhage proved to be a sac, formed by the uterine portion of the left Fallopian tube and the wall of the uterus, which had grown outwardly to about the size of a walnut, and then ruptured anteriorly. Chorion villi were distinctly visible in the sac. The opening of the tube into the sac had become obliterated. There was evidence of a previous partial rupture, in the shape of a small hæmatocele, on the posterior aspect of the sac. The fœtus had escaped into the abdominal cavity, and was unfortunately lost. The left ovary was closely attached to the left side of the uterus by old bands of lymph, and contained several cysts. The right ovary was normal, and contained a corpus luteum. The uterus was enlarged, and its lining membrane was red and thickened, forming a distinct decidua, that could be easily detached. The bladder was healthy, but contained no urine. The abdominal organs were healthy, but very anæmic.

With regard to the cause of the arrest of the ovum in that particular spot, I may remark that nothing existed in the Fallopian tube or uterus, in the shape of polypus or fibroid, to cause obstruction, but that there were plenty of adhesions on the left side, matting the uterus, Fallopian tube and ovary together, altering their relative positions, and, possibly, causing obstruction. Yet the presence of a corpus luteum in the right ovary, coupled with the cystic condition of the left, would point to the theory of transmigration of the ovum as being the most probable explanation of the phenomenon."—*British Medical Journal.*

